# Designing and evaluation of Dot-ELISA for diagnosis of *Fasciola *infection in cattle

**Published:** 2014

**Authors:** Jafar Arjmand, Bijan Esmaeilnejad, Mohammad Hossein Razi Jalali, Masoud Ghorbanpoor, Seyyed Meysam Abtahi Froushani

**Affiliations:** 1 *Department of Pathobiology, Faculty of Veterinary Medicine, Urmia University, Urmia, Iran;*; 2 * Department of Pathobiology, Faculty of Veterinary Medicine, Ahvaz University, Ahvaz, Iran; *; 3 *Department of Microbiology, Faculty of Veterinary Medicine, Urmia University, Urmia, Iran.*

**Keywords:** Cattle, Dot-ELISA, Fasciolosis

## Abstract

Fasciolosis is a disease caused by liver fluck of the genus of *Fasciola*. Diagnosis of fasciolosis has been challenging for a long period due to low sensitivity of the coprological diagnostic method. In this study, an in-house Dot-ELISA method; using excretion–secretory (ES Ag) and Crude (Cr Ag) antigens of *Fasciola *was described for diagnosis of fasciolosis in cattle. For this purpose, the sera specimens of slaughtered cattle were taken and examined for *Fasciola *infection. Sera from two groups of cattle, one infected with *Fasciola* (n = 60) and the other non-infected with *Fasciola* (n = 60), were used in the Dot- ELISA test. All sera were tested and evaluated. Except specificity, other parameters such as, sensitivity, accuracy, positive and negative predictive values of Dot- ELISA with ES Ag were better than those of Dot- ELISA with Cr Ag. In conclusion, excretory–secretory antigen dependent Dot-ELISA can be used as a reliable sero-diagnostic test for *Fasciola *infection in cattle.

## Introduction

*Fasciola* causes considerable economic loss to the meat industry. Clinical signs and symptoms appear three weeks post-infection. Furthermore, early diagnosis is not possible because eggs are not found in faeces until flukes reach maturity, usually between 10 and 14 weeks after infection, hence, fasciolosis coprological diagnostic method has presented a challenge. Therefore, a more accurate diagnostic test design may be valuable.^1^

Serological techniques have been evaluated for the diagnosis of fasciolosis.^2^ Antibody detection assays are overwhelmingly the preferred method for immune diagnosis of fasciolosis. The reasons include the relative simplicity of the assays and early sero-conversion (usually 1-2 weeks).^3^ Many serological methods, thus far, have been challenged to diagnose human and animal fasciolosis. However, most of them have varied in specificity and sensitivity due to differences in materials and methods, and of course, may owing to differences in the nature of the parasite, being utilized to prepare antigen. Of these methods, hemaglutination (HA), indirect fluorescence antibody test (IFAT), immunoperoxidase (IP), counter electrophoresis (CEP), enzyme-linked immunosorbent assay (ELISA) and Dot-ELISA could be mentioned. The latter technique, due to simplicity, having the prospect to be implanted in field trials, is regarded as an important method in its turn.^4^^-^7 The purpose of this study was to describe an in-house Dot- ELISA using two extracted *Fasciola* (crude and excretory-secretory) antigens for diagnosis of fasciolosis in cattle.

## Materials and Methods

**Sample collection. **Cattle at Ahvaz slaughter houses were slaughtered and examined macroscopically for the presence of mature and immature *Fasciola *flukes in their livers, bile ducts, and gall bladder according to the method of Anderson *et al*.^1^ The liver was then sliced into strips of about 1 cm in thickness and soaked in normal saline for 1 hr. Flukes emerging from the cut bile ducts were put into the small glass and each sliced strip was thoroughly squeezed from end-to-end, washed in saline, and discarded. The contents of the basin were sieved; placed in a Petri dish; and the adult, immature, and cut pieces of flukes were added to the glass. Identification of the live flukes was performed according to Andrews.^8^ The number of *Fasciola *was counted as described by Anderson *et al*.^1^

Examined cattle were divided into *Fasciola*-infected and *Fasciola*-free groups. Blood samples were allowed to clot on the bench at room temperature at an inclined position for 2 hr, centrifuged at 3,000 rpm for 30 min. The serum samples were obtained and stored at −20 ˚C until tested. A total number of 60 serum samples from *Fasciola*-infected and 60 serum samples from *Fasciola*-free cattle were collected.

**Preparation of crude worm antigen.** *Fasciola *crude worm antigen (Cr Ag) was prepared as described by Oldham and Williams with some modification.^9^ Briefly, adult *Fasciola* flukes were washed three times in phosphate-buffered saline (PBS; 0.01 mM, pH 7.4), drained and freeze- dried for 24 hr at −70 ˚C. The dried flukes were ground into a fine powder and suspended in PBS, then homogenized in a high-speed mixer for 15 min and were stored overnight at 4 ˚C. After centrifugation at 3,000 rpm for 15 min, the supernatant was filtered and sterilized by passing through 0.45 and 0.22 μm filters, aliquotted, and stored at −20 ˚C until assayed.^9^

**Preparation of** ***Fasciola*** **excretory/secretory antigens.** *Fasciola* excretory/secretory antigen (ES Ag) was prepared according to Simsek *et al*.^7^ Briefly, adult *Fasciola* helminthes were washed several times in 0.01 mM PBS (pH 7.4). The specimens were incubated in PBS (5 flukes per 10 mL) at 37 ˚C and 5% CO_2_ for 6 hr. PBS containing E/S products were centrifuged at 10,000 rpm for 30 min at 4 ˚C. The supernatant was filtered through filter with 0.22 μm in size. The products were dialyzed against distilled water for 24 hr, aliquotted, and stored at −20 ˚C until analysed.^10^

**Measurement of protein concentration in prepared antigens. **Protein concentration of each antigen (Cr and ES) was measured according to the method described by Lowry *et al*.^11^

**Dot-ELISA method. **Dot-ELISA was conducted as described earlier, with some modification.^12^ Optimal serum, antigen, and bovine anti-IgG peroxidase conjugate (Abcam) concentrations were determined after preliminary checkerboard titration. Briefly, 1 μg of *Fasciola *ES and Cr antigen was dotted on nitrocellulose membrane discs and allowed to be dried thoroughly. The discs were placed into flat bottom micro plate wells. Non-specific binding sites were blocked by addition of skimmed milk (Merk, Darmstadt, Germany). Blocking solution was then aspirated off and antigen disks were washed by shaking (three times, 10 min each) with 0.05% Tween 20 (Riedel de Haen AG, Seelze, Germany). Amount of 100 μL of 1:5 dilution of serum was added to each disk before incubation for 1 hr at room temperature. After washing (see above) 100 μL of a 1:5,000 dilution of anti-bovine IgG peroxidase conjugate was added to each disk and the plate was incubated for 1 hr at room temperature. After washing, 100 μL substrate including tetra-methyl benzedine and H_2_O_2_ (Serotech, Seoul, South Korea) was added into each well and incubated for 25 min at room temperature. The development of a deep brown color dot on disks when compared with negative serum control was considered to be evidence of positivity.^12^^,^^13^

In addition to *Fasciola*, two recent parasites are the main causes of liver parasite infestation in ruminant. Therefore, serum samples of positive infected cattle with dicrocoeliasis and hydatidosis were used for evaluation of cross reactivity.^12^

**Evaluation of Dot-ELISA. **Results of the Dot-ELISA using different antigens of *Fasciola* to detect IgG against *Fasciola* in sera were evaluated comparing to the results of presence or absence of flukes in their livers. This was taken as a gold standard and the diagnostic sensitivity, specificity, precision, positive predictive value and negative predictive value of the assay were calculated as follow:^13^^,^^14^

$\mathrm{Sensitivity}=\frac{\mathrm{TP}}{\mathrm{TP}+\mathrm{FN}}\times 100$

$\mathrm{Specificity}=\frac{\mathrm{TN}}{\mathrm{TN}+\mathrm{FP}}\times 100$

$\mathrm{Precision}=\frac{\mathrm{TN}+\mathrm{TP}}{N}\times 100$

$\mathrm{Positive Predictive value}=\frac{\mathrm{TP}}{\mathrm{TP}+\mathrm{FP}}\times 100$

$\mathrm{Negative Predictive value}=\frac{\mathrm{TN}}{\mathrm{TN}+\mathrm{FN}}\times 100$

where *TP* is true positive, *TN* is true negative, *FP* is false positive, *FN* is false negative and *N* is total sample.

## Results

Total protein of Cr and ES antigens were 900 and 85 mg dL^-1^, respectively. The results of both examination of livers for the presence or absence of liver fluke and the corresponding results from the Dot-ELISA using Cr and ES antigens were obtained and presented in Table 1.

**Table 1:** Results of examination of cattle livers for liver flukes and Dot-ELISA on their sera using Cr and ES antigens, (* n = 60).

**Antigen type**	**Result**	**Infected** *		Non-Infected*
**Cr Ag**	**Positive**	54	9	
	**Negative**	6	51	
**ES Ag**	**Positive**	56	7	
	**Negative**	4	53	

The diagnostic sensitivity, specificity, precision, positive predictive value and negative predictive value percentages of Dot-ELISA using Cr and ES antigens for diagnosis of *Fasciola* infection in cattle were calculated and recorded in Table 2. 

Cross-reactions were not detected in Dot-ELISA by ES antigen but in ½, ⅓, ¼ and ⅕ μg of dilution Cr antigen detected with dicrocoeliasis and hydatidosis.

**Table 2:** The diagnostic sensitivity, specificity, and precision, positive predictive value and negative predictive value percentages of Dot-ELISA using Cr and ES antigens for diagnosis of *F. gigantica *infection in cattle

**Antigen type**	**Cr Ag (%)**	**ES Ag (%)**
**Sensitivity **	90.00	93.33
**Specificity **	85.00	88.33
**Precision **	87.50	90.83
**Positive predictive value**	85.71	88.88
**Negative predictive value **	89.47	92.98

## Discussion

Fasciolosis historically has been a disease of ruminants worldwide and caused economic losses in the animal husbandry industry.^2^ Dot-ELISA has been considered as one of the valuable methods in diagnosis of different parasitological diseases like fasciolosis, toxoplasmosis, shistosomiasis, hydatidosis and cysticercosis in a range of definitive hosts.^4^ In the present study, we designed a very rapid and reliable immunoassay for screening of *Fasciola* in cattle herds. This method may be simply employed in the clinic or fields and gives useful information in a short time. 

The ELISA, especially the FAST ELISA method, is an excellent screening test, followed by the Western immuno-blot as the confirmatory test.^15^^-^^18^ However, ELISA is a very sensitive test, but this assay is a time consuming method and needs some instruments and reagents. In this study, three positive bovine blood samples of dicrocoeliasis and three positive bovine blood samples of hydatidosis were positive by Dot-ELISA at ½, ⅓, ¼ and ⅕ μg dilution of crude antigen of *Fasciola* but in ES antigen, cross-reaction was not detected. The false negative results may be attributed to modulation of the host immune response by liver flukes as reported by Anderson *et al*.^1^

Higher sensitivity for the diagnosis of bovine fasciolosis was obtained using ES Ag (93.33%) compared with Cr Ag (90.00%). High specificity (88.33%) was recorded when ES Ag was used in Dot-ELISA for diagnosis of *Fasciola* infection compared with 85.00% using Cr Ag. We propose that this may be attributed to the more numerous antigenic components of Cr Ag compared with those of ES Ag leading it to be more sensitive and less specific than ES Ag. Higher precision (90.83%) was obtained when using Dot-ELISA with ES Ag for diagnosis of bovine fasciolosis compared with using Cr Ag (87.50%). Using ES Ag in Dot-ELISA gives high-accuracy rates. Recently, native cathepsin-L cysteine proteinase was purified from the excretory secretory products of *Fasciola* and applied for sero-diagnosis of *Fasciola* infection in buffaloes using Dot-ELISA. The results demonstrated that cathepsin-L cysteine proteinase based Dot-ELISA achieved 90.00% sensitivity and 100% specificity.^19^^,^^20^ A previous document showed that sandwich-Dot- ELISA had better sensitivity and specificity than S-ELISA for both stool and serum, and may be used as a rapid screening test in field.^21^

In conclusion, ES Ag was the best coating antigen in Dot-ELISA for the sero-diagnosis of fasciolosis in cattle due to its high sensitivity, specificity, and precision rates. It seems that in heavy infected bovine with *Fasciola*, the possibility of sero-diagnosis was increased.
